# Dynamics of Rare Earth Element Uptake and Partitioning in Two Soybean Cultivars Under Background Soil Conditions

**DOI:** 10.3390/plants15162485

**Published:** 2026-08-16

**Authors:** Yaofu Chen, Jilong Lu, Xinyun Zhao, Peng Lu, Jiaxuan Cui, Kaiyu Zhang, Jiayu Qu, Yaru Hou

**Affiliations:** 1 College of Geoexploration Science and Technology, Jilin University, Changchun 130026, China; yaofu24@mails.jlu.edu.cn (Y.C.); zhaoxy15@jlu.edu.cn (X.Z.); lupeng@jlu.edu.cn (P.L.);; 2 School of Geomatics and Prospecting Engineering, Jilin Jianzhu University, Changchun 130118, China; houyaru@jlju.edu.cn

**Keywords:** rare earth elements (REEs), source-to-sink dynamics, developmentally-gated translocation, redox-mediated bio-fractionation, genotype-specific compartmentalization, crop elemental homeostasis

## Abstract

Background: Rare earth elements (REEs) are present in agricultural ecosystems, but their baseline soil–plant transfer dynamics require systematic understanding. This study investigated the ‘source-to-sink’ biogeochemical distribution of REEs in soybean under background soil conditions. Methods: We characterized REE concentrations and fractionation patterns in the composite surface soil samples and analyzed two soybean cultivars (‘Jiyu 201’ (JY) and ‘Ping’an 16’ (PA)). A seven-stage sampling scheme (20–80 days post-sowing) using whole-plant samples was employed to evaluate temporal concentration dynamics, followed by discrete organ-level analysis at maturity. Results: The composite surface soil samples exhibited a light REE (LREE)-enriched signature with negative cerium (Ce) and europium (Eu) anomalies. While plants consistently mirrored the soil’s negative Eu anomaly, an observable shift toward a positive Ce anomaly was detected in plant tissues during the 45–65 days post-sowing window. REE concentrations were markedly higher in roots than in aerial tissues, indicating strong root-associated retention and limited acropetal translocation; however, the root values include both internalized and surface-associated fractions. Acropetal transport was accompanied by preferential LREE enrichment. Final organ-level partitioning was highly genotype-dependent: the indeterminate cultivar PA accumulated higher REEs in beans, whereas the sub-determinate cultivar JY retained them primarily in vegetative stems. Conclusions: Under background soil conditions, REE uptake and partitioning in soybean exhibit dynamic and genotype-dependent patterns. The observed shift in the Ce anomaly presents a notable biogeochemical phenomenon. Establishing these baseline dynamics is vital for evaluating crop elemental homeostasis and utilizing REEs as biogeochemical tracers.

## 1. Introduction

The biogeochemical cycling and environmental fate of rare earth elements (REEs)—comprising the 15 lanthanides alongside scandium (Sc) and yttrium (Y)—have received increasing attention in terrestrial ecosystems [1,2]. As emerging trace contaminants and unique geochemical proxies, REEs participate in complex soil–plant transfer processes governed by pedogenic weathering, soil pH, redox potential, organic matter, and rhizosphere exudation [3,4,5]. During pedogenesis and plant uptake, individual REEs undergo systematic fractionation between light REEs (LREEs, La–Eu) and heavy REEs (HREEs, Gd–Lu), frequently exhibiting distinct redox-sensitive anomalous behaviors (such as cerium, Ce, and europium, Eu, anomalies) that reflect environmental redox conditions and parent material lithology [6,7,8,9]. With the expanding industrial applications of REEs and their incidental releases through agricultural micro-fertilizers and phosphate inputs, elucidating the soil continuity, bioaccumulation, and trophic transfer of REEs has become essential for environmental safety, crop food safety, and sustainable agricultural production [10,11].

From a plant ionomics and physiological standpoint, REEs are not classified as essential plant nutrients, yet they exhibit significant biological activity within plant cellular networks [12]. Owing to their chemical similarity and ionic radii comparable to essential divalent cations (e.g., Ca^2+^) and trivalent metals (e.g., Fe^3+^ and Al^3+^), REE ions can co-opt endogenous nutrient transport pathways across plant membranes [1,10,12]. At low background or exposure concentrations, REEs frequently display a hormetic dose-response, stimulating seed germination, root elongation, photosynthetic efficiency, and antioxidant enzyme activities in major crops [3,13,14]. Within the conceptual framework of the plant ionome—defined as the total inorganic elemental composition of an organism—understanding how plants regulate non-essential trace elements alongside essential macronutrients and micronutrients provides fundamental insights into crop elemental homeostasis [15,16]. In leguminous crops such as soybean (*Glycine max* L.), specific REE exposures have been shown to modulate seedling physiological performance, reactive oxygen species signaling, and nutrient acquisition [14,17,18].

Despite extensive laboratory, hydroponic, and pot-based investigations, a systematic, field-based understanding of the ‘source-to-sink’ biogeochemical dynamics of REEs under natural soil background conditions remains incomplete [19,20]. Major observational gaps persist regarding: (1) how temporal REE uptake, accumulation, and elemental fractionation dynamics dynamically evolve across consecutive phenological stages over a complete lifecycle; (2) how specific plant genetic traits and growth habits govern the terminal allocation of REEs among discrete vegetative and reproductive organs; and (3) how redox-sensitive anomalies (such as Ce shifts) dynamically unfold at the root–soil interface during key phenological transitions. Modern crop cultivars with distinct growth habits (e.g., determinate vs. indeterminate soybeans) exhibit fundamental differences in canopy architecture, transpirational pull, root system depth, and sink-source nutrient allocation demands [21,22]. These genetic and biophysical traits may fundamentally alter REE bioaccumulation profiles and vascular mobility, yet such genotype-dependent interactions under natural background conditions remain poorly constrained.

To address these critical knowledge gaps without the confounding artifacts of artificial high-dose REE spiking, this study investigated the baseline soil–plant REE continuity in the typical black soil (Mollisols) region of Northeast China, a principal national soybean production base [23,24,25,26,27]. We comparatively evaluated two soybean cultivars possessing contrasting biological traits: ‘Jiyu 201’ (JY; a sub-determinate, early-maturing cultivar) and ‘Ping’an 16’ (PA; an indeterminate, taller cultivar). Our specific objectives were to: (I) characterize the geochemical baseline and fractionation signature of the natural background soil REE source; (II) track temporal concentration dynamics of 14 individual REEs (La–Lu, excluding radioactive Pm), soil-to-plant transfer factors (TF), and redox-mediated fractionation shifts (including Ce and Eu anomalies) across seven consecutive developmental stages (20–80 days post-sowing); and (III) determine final organ-level partitioning (roots, stems, pods, beans) at full harvest maturity. Establishing these baseline dynamics is vital for advancing crop ionomic research, optimizing micronutrient management within agroecosystems, and evaluating REEs as natural biogeochemical tracers of elemental cycling within agricultural ecosystems.

## 2. Results

### 2.1. Geochemical Signature of REEs in Soils

#### 2.1.1. REE Concentrations and LREE/HREE Ratios

The composite surface soil samples exhibited a consistently weakly acidic character, with pH values ranging from 6.28 to 6.56 (mean 6.40 ± 0.09; Supplementary Table S2). The total REE concentration (ΣREE) in the surface soil samples ranged from 43.44 to 245.91 mg/kg, with a mean of 130.24 mg/kg and a high coefficient of variation (52.04%). Light REEs (LREE) were predominant, with ΣLREE concentrations averaging 116.19 mg/kg, while heavy REEs (HREE) were less abundant (mean: 14.04 mg/kg). Consequently, the ΣLREE/ΣHREE ratio varied from 5.82 to 13.29 (mean: 8.44). Chondrite-normalized ratios further elucidated this fractionation: the (La/Yb)_N_ ratio ranged from 9.04 to 19.74, while the (La/Sm)_N_ ratio (3.40–4.88) and the (Gd/Yb)_N_ ratio (2.12–4.46) were also calculated (Supplementary Tables S1).

#### 2.1.2. Relative Abundance and Fractionation Patterns

**Figure 1:**
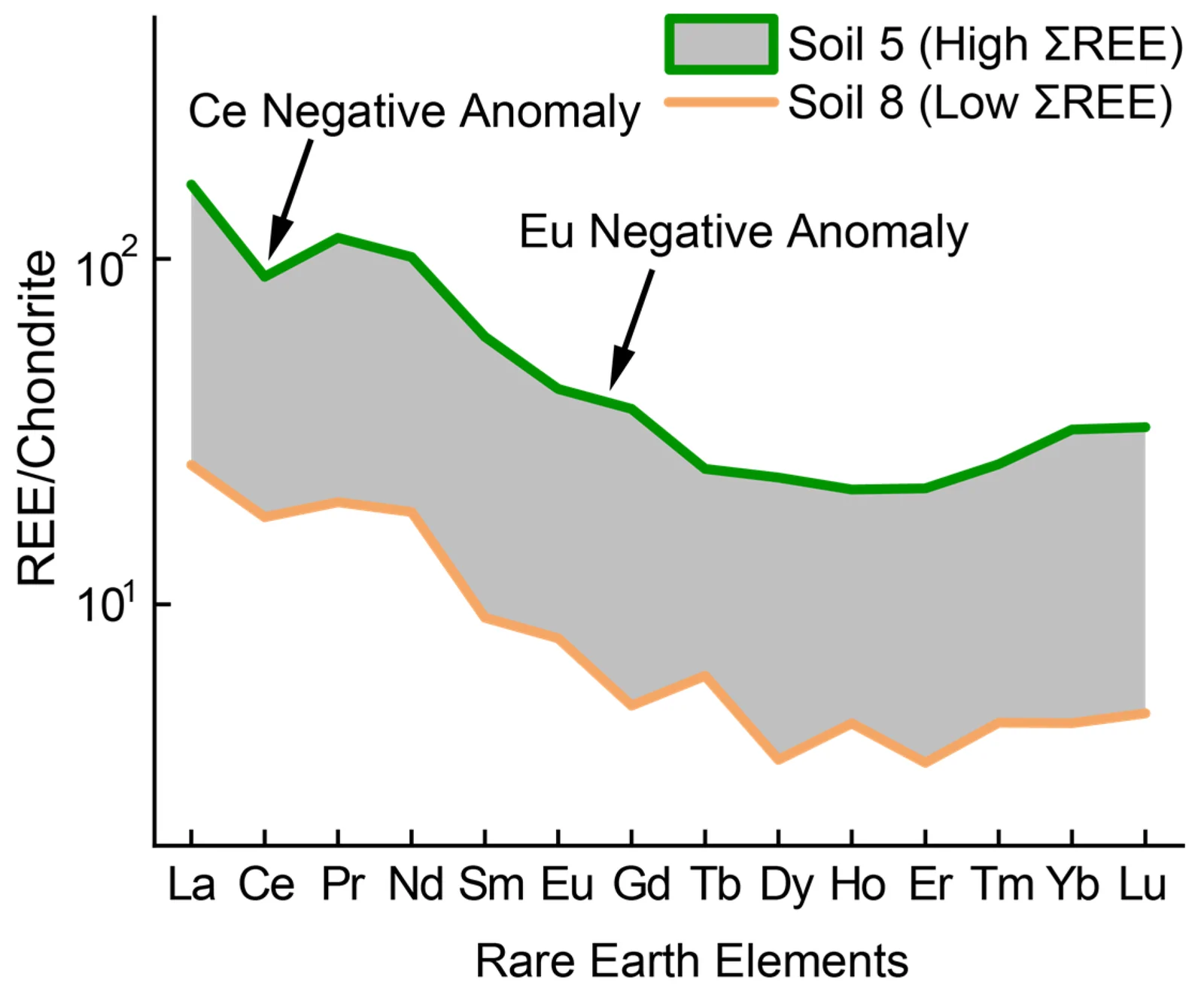
Chondrite-normalized fractionation patterns of rare earth elements (REEs) in the surface soil samples. The green and orange solid lines represent Soil 5 (High ΣREE) and Soil 8 (Low ΣREE), respectively, illustrating the upper and lower boundaries of the observed REE concentration range (grey shaded area). Black arrows highlight the characteristic negative anomalies for cerium (Ce) and europium (Eu). The detailed chondrite-normalized values and raw elemental concentrations are provided in Supplementary Table S2.

To illustrate the average compositional structure of REEs in the soils, the mean proportion of each individual REE relative to the ΣREE across all 11 samples is presented in Table 1 (Detailed raw concentrations for all 11 soil samples are provided in Supplementary Table S2). The data reveal a distinct and consistent hierarchical pattern of relative abundance. Specifically, cerium (Ce) accounts for the largest share of the ΣREE, followed by neodymium (Nd) and lanthanum (La), while thulium (Tm) consistently represents the smallest fraction. To clearly illustrate the dominant fractionation trends while minimizing visual complexity, the chondrite-normalized REEs distribution patterns for three representative samples are presented in the Masuda–Coryell diagram (Figure 1). These samples were selected to span the observed concentration range, representing low (Soil 8) and high (Soil 5) total REE contents. Despite the wide variation in absolute concentrations, all three samples exhibit a consistent fractionation pattern. As highlighted in the figure, this pattern is characterized by slight but distinct negative anomalies for both cerium (Ce) and europium (Eu).

### 2.2. Bioaccumulation and Fractionation Dynamics of REEs During Soybean Growth

#### 2.2.1. Developmentally-Associated Shift of the Cerium Anomaly

**Figure 2:**
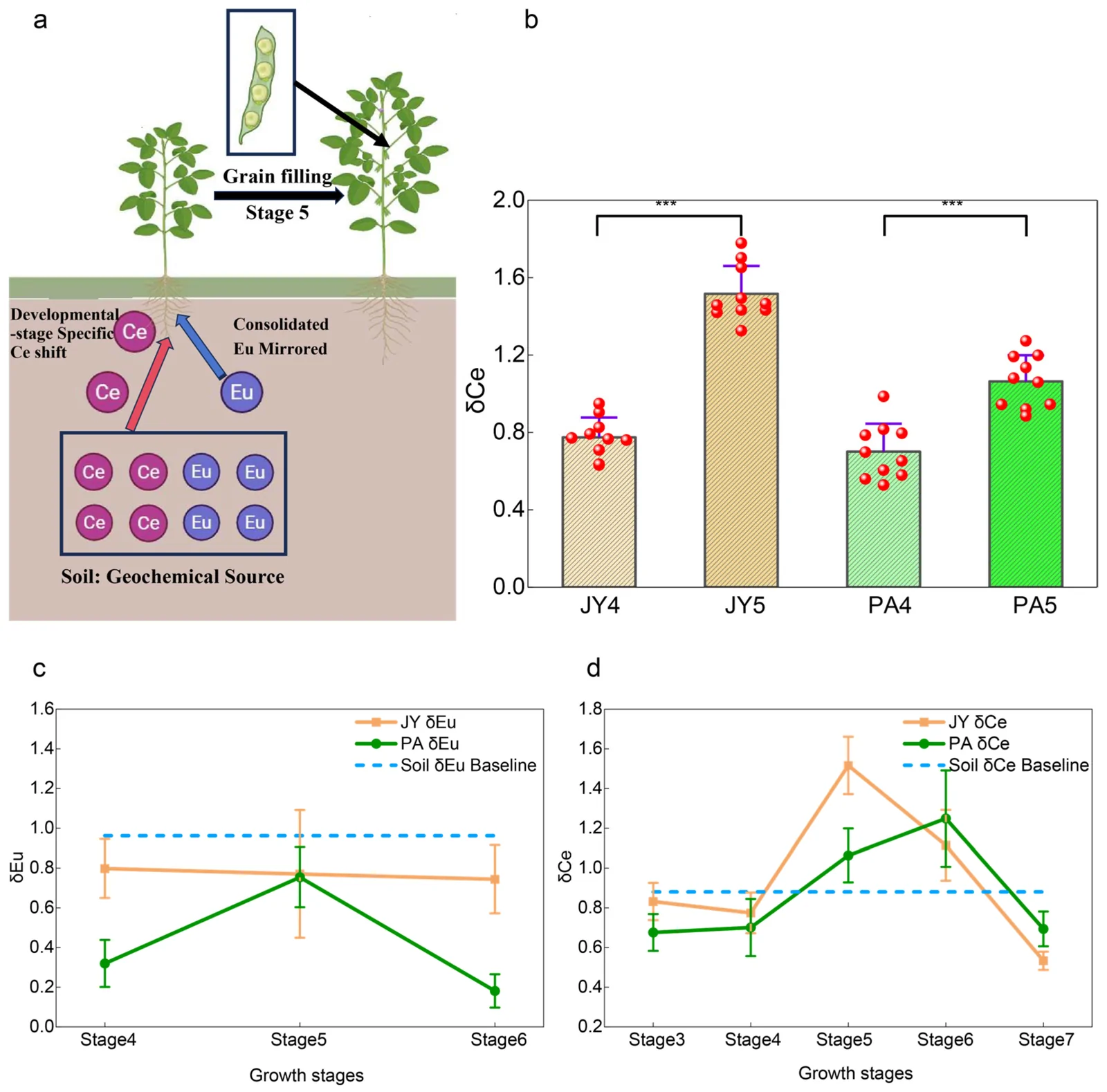
Contrasting temporal patterns of cerium (Ce) and europium (Eu) anomalies in soybean plants. (**a**) A conceptual model illustrating the observed developmental-stage-specific shift of the Ce anomaly alongside the consistently mirrored soil Eu anomaly. (**b**) A direct comparison of the Ce anomaly (δCe) between Stage 4 and Stage 5, confirming a significant positive shift in δCe between these two stages. (**c**,**d**) Temporal dynamics of Eu and Ce anomalies (δEu and δCe) across distinct growth stages, showing the divergence of these anomalies during the grain-filling period (Stage 5). Orange lines and bars represent the JY cultivar, while green lines and bars represent the PA cultivar. The blue dashed line indicates the geochemical baseline of the soil. Data are presented as mean ± SD (*n* = 10 for JY3 to JY7 and PA4 to PA5, *n* = 6 for PA3, PA6 and PA7). Statistical significance was determined using a two-tailed Student’s *t*-test (***, *p* < 0.001). The underlying dataset for temporal dynamics across the five developmental stages is detailed in Supplementary Tables S3–S12. (Detailed data for the soil baseline are available in Supplementary Table S2).

The geochemical baseline of the composite surface soil samples exhibited a consistent negative anomaly for both cerium (Ce) and europium (Eu). A negative Eu anomaly consistent with the soil was consistently observed in plant tissues across all growth stages. In contrast, the accumulation pattern of Ce was highly dynamic. While mirroring the soil’s negative signature in early growth stages, a distinct shift occurred during the subsequent reproductive period: a positive Ce anomaly was observed in the plant tissues of both cultivars during a specific 45–65 days post-sowing window (Figure 2).

#### 2.2.2. Identification of a Peak Period for REE Accumulation

**Figure 3:**
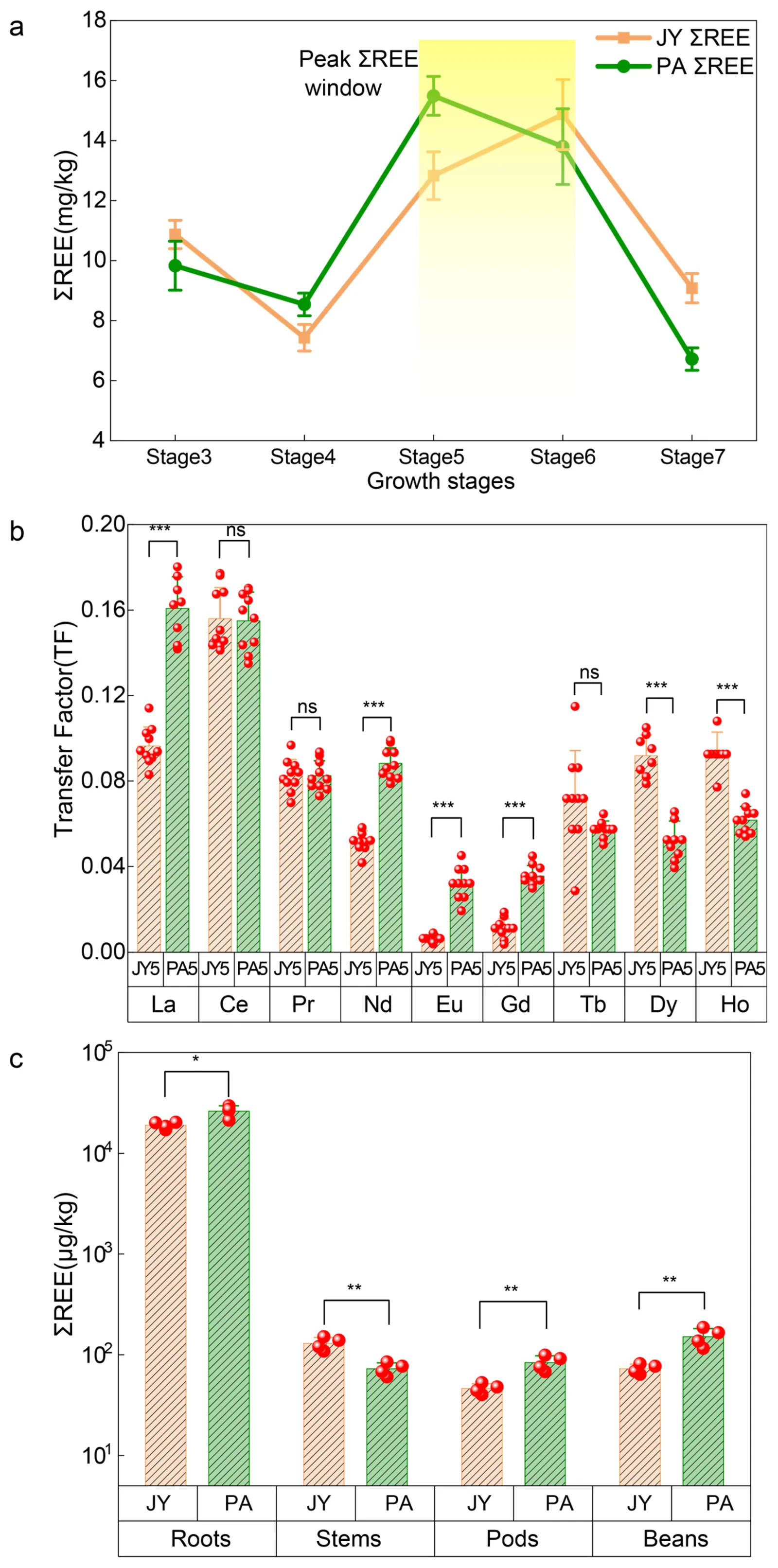
Dynamics, translocation, and organ-specific distribution of rare earth elements (REEs) in JY and PA soybean cultivars. (**a**) Temporal dynamics of ΣREE concentrations in soybean plants across growth stages, showing an accumulation peak between 45 and 65 days after sowing (highlighted by the yellow shaded area). (**b**) Soil-to-plant transfer factors (TF) of REEs in JY and PA soybean cultivars between 45 and 55 days after sowing, comparing the TF values of specific light and heavy REEs between the two cultivars. (**c**) Final ΣREE concentrations in discrete plant organs at maturity, showing the distribution in vegetative and reproductive tissues. Orange colors represent the JY cultivar, while green colors represent the PA cultivar. In panels (**b**,**c**), data are presented as striped bars with red dots indicating individual biological replicates. Data are shown as mean ± SD (for panel (**a**), *n* = 10 for JY3 to JY7 and PA4 to PA5, and *n* = 6 for PA3, PA6, and PA7; for panel (**b**), *n* = 10; for panel (**c**), *n* = 4). Statistical significance was determined using a two-tailed Student’s *t*-test (***, *p* < 0.001, **, *p* < 0.01, *, *p* < 0.05 and ‘ns’ denotes no significant difference). The underlying raw datasets for the temporal dynamics across distinct developmental stages (**a**) and the subsequent TF calculations (**b**) are provided in Supplementary Tables S3–S12. The detailed concentrations and descriptive statistics for the organ-specific distribution at maturity (**c**) are available in Supplementary Tables S17 and S18.

The temporal dynamics of ΣREE accumulation were investigated throughout the soybean life cycle. As depicted in Figure 3a, ΣREE concentrations in both cultivars were not static, but followed a distinct developmental pattern. A peak accumulation period was identified between 45 and 65 days after sowing, a period corresponding to the reproductive stages from late pod-filling to pre-bulging. Within this window, ΣREE concentrations peaked at 14.87 mg/kg and 15.49 mg/kg for the JY and PA cultivars (Detailed raw concentrations for ΣREE concentrations are provided in Supplementary Tables S7, S8, S13, S14 and S15), respectively. This trend was primarily driven by the LREE, whose temporal concentration profile closely mirrored that of the ΣREE.

#### 2.2.3. Soil-to-Plant Transfer Dynamics Across Primary Growth Stages Evaluated by TF

To quantitatively evaluate the temporal dynamics and cultivar-specific differences in soil-to-plant REE transfer, TF values were calculated for all seven developmental sampling stages. For clarity and to emphasize the primary active growth period, detailed TF values for Stages 3–7 are presented in Table 2, whereas the corresponding values for the two developmental boundary stages, Stage 2 and Stage 8, are provided in Supplementary Table S16. Across Stages 3–7, TF values exhibited pronounced element-specific and developmental-stage-dependent variation rather than a uniform temporal pattern. In JY, Ce, Dy, Ho, and Er reached their highest TF values within this five-stage interval at Stage 5, whereas La, Nd, and Gd reached their highest values at Stage 6, and Pr, Sm, Eu, Tb, Tm, Yb, and Lu reached their highest values at Stage 7. In PA, the timing of the highest TF values within Stages 3–7 was similarly heterogeneous: Ce, Nd, Eu, and Gd reached their maxima at Stage 5; La, Ho, and Yb at Stage 6; Sm, Tb, and Dy at Stage 7; Pr at Stage 3; and Er, Tm, and Lu at Stage 4. These temporal patterns demonstrate that soil-to-plant REE transfer varied substantially among individual elements and developmental stages, with additional differences between the two cultivars (Table 2).

Cultivar-specific differences were particularly evident at Stage 5 (50 days post-sowing), which falls within the reproductive accumulation window. At this stage, PA exhibited significantly higher TF values than JY for La (0.161 vs. 0.096), Nd (0.088 vs. 0.052), Eu (0.034 vs. 0.006), and Gd (0.035 vs. 0.011) (all *p* < 0.001), whereas JY exhibited significantly higher TF values for Dy (0.093 vs. 0.054) and Ho (0.093 vs. 0.055) (both *p* < 0.001). In contrast, no significant inter-cultivar differences were detected for Ce, Pr, or Tb at this stage (Figure 3b). These contrasting patterns indicate that cultivar effects on REE transfer were element-specific rather than uniform, further supporting genotype-dependent differences in soil-to-plant REE transfer during the reproductive period.

#### 2.2.4. Inter-Elemental Correlations During Peak Accumulation

**Figure 4:**
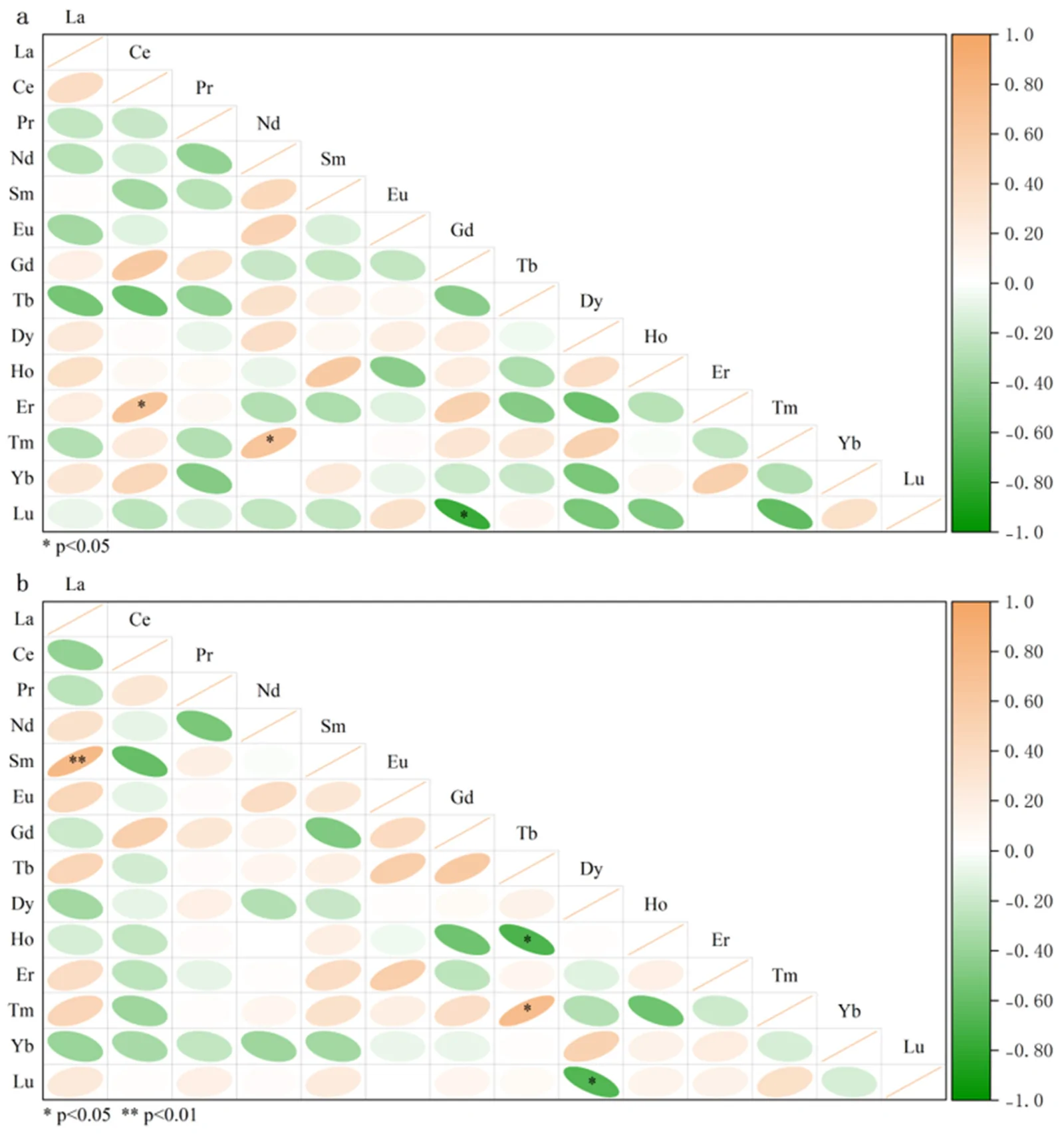
Pearson correlation heatmap of 14 rare earth elements in (**a**) JY and (**b**) PA soybean plants between 45 and 55 days after sowing. The color gradient represents the strength and direction of the correlation coefficients (*r*), with orange indicating positive correlations and green indicating negative correlations. Statistical significance is denoted by asterisks (*, *p* < 0.05, **, *p* < 0.01). Correlation analyses were performed across ten independent biological replicates for each cultivar (*n* = 10). The underlying raw concentration datasets utilized for these analyses are provided in Supplementary Tables S7 and S8.

To further explore the coordinated behavior of REEs during the peak accumulation period (45–55 days after sowing), Pearson correlation analyses were conducted among the 14 individual REEs for both cultivars (Figure 4). The heatmaps illustrate distinct intra-group and inter-group relationships. Strong positive correlations (indicated by orange gradients) were predominantly observed within the LREE group and within the HREE group, respectively. In contrast, correlations between individual LREEs and HREEs frequently exhibited weaker or negative relationships (indicated by green gradients). While this broader correlation pattern was common to both cultivars, the specific element pairs showing statistical significance varied between JY and PA, providing further quantitative data on cultivar-specific elemental accumulation during this specific period.

### 2.3. Organ-Level Distribution and Fractionation of REEs in Mature Soybean

#### 2.3.1. High Total Root-Associated REE Concentrations

Analysis of REE concentrations in the discrete organs of mature JY and PA soybean plants revealed a pronounced concentration contrast between below-ground and above-ground tissues. Roots exhibited the highest total root-associated REE concentrations among all analyzed organs, with ΣREE concentrations of 18.94 mg/kg in JY roots and 26.14 mg/kg in PA roots. These measured root-associated concentrations were approximately two to three orders of magnitude higher than those in the aerial organs, which ranged from 46.23 to 129.67 μg/kg (Figure 3c; Supplementary Tables S17 and S18). However, because chemical desorption was not performed prior to root digestion, the measured root concentrations represent the total root-associated REE pool, encompassing both internally accumulated REEs and fractions strongly adsorbed to the root surface or apoplast. Therefore, the magnitude of true internal root accumulation cannot be quantitatively distinguished from surface-associated fractions in the present dataset. Nevertheless, the markedly lower REE concentrations observed in aerial tissues relative to roots are consistent with restricted root-to-shoot translocation, although the extent of biological root sequestration should be interpreted with appropriate caution.

#### 2.3.2. Genotype-Dependent Allocation and Fractionation in Aerial Organs

Within the aerial parts of the plant, REE distribution varied between the two cultivars. In the JY cultivar, the highest ΣREE concentration was observed in the stems (129.67 μg/kg), followed by the beans (72.67 μg/kg) and pods (46.23 μg/kg). In contrast, the majority of translocated REEs accumulated in the beans of the PA cultivar (150.92 μg/kg), which exhibited higher concentrations than the pods (83.89 μg/kg) and stems (72.50 μg/kg).

Systematic elemental fractionation was observed along the transport path. In all organs of both cultivars, LREE were preferentially enriched over HREE, as indicated by ΣLREE/ΣHREE ratios consistently greater than 1. The degree of this fractionation, however, intensified from root to shoot, following the general trend: pods ≥ beans > stems > roots. This trend was further corroborated by (La/Yb)_N_ ratios, which ranged from 5.00 to a maximum of 163.69, confirming substantial LREE enrichment. Furthermore, the degree of fractionation within the LREE group (mean (La/Sm)_N_ = 13.99) was significantly more pronounced than fractionation within the HREE group (mean (Gd/Yb)_N_ = 3.06) (Supplementary Table S18).

## 3. Discussion

### 3.1. Soil as the Geochemical Source: A Constrained Reservoir Governing Bioavailability

The geochemical signature of the composite surface soil provides a field-level geochemical reference for understanding in-planta REE accumulation. The observed mean ΣREE concentration (130.24 mg/kg) aligns with typical Mollisols in Northeast China (141.45 mg/kg in subsurface black soils [28]) but remains lower than the Chinese national soil background average (139.0 mg/kg) and significantly lower than highly weathered acidic soils in Southern China (346.16 mg/kg [29]). The distinct LREE-enriched pattern (mean ΣLREE/ΣHREE = 8.44) and the slight negative Eu anomaly are characteristic features of upper continental crust derived from weathered felsic parent rocks, which exert a primary geochemical control over the elemental composition of the soil–sediment continuum [30]. During pedogenesis, soil organic matter and clay minerals preferentially stabilize specific REEs, shaping the ultimate bioavailable pool [31,32]. The negative Ce anomaly may further suggest an influence from past marine environments during soil genesis [33].

A critical area for future research lies in a more granular characterization of the “bioavailable pool” itself, because total concentration is not a direct proxy for bioavailability. The fraction of REEs accessible to roots is governed by elemental speciation and rhizosphere dynamics, particularly the exudation of low-molecular-weight organic acids (LMWOAs) by plant roots [19]. In our study, the composite surface soil indicated a weakly acidic field-level soil background (mean pH 6.40 ± 0.09; Supplementary Table S2), which thermodynamically favors the chemical stability of dissolved trivalent REE ions in the soil solution relative to alkaline conditions [6], Building upon this favorable geochemical baseline, plant root growth actively modulates this local soil solid-phase availability; as soybean roots absorb major nutrient cations (such as K^+^ and Ca^2+^), the concomitant extrusion of H^+^ and LMWOAs locally enhances the dissolution and root-surface sorption of REEs over time [34,35]. While our baseline data establishes the total elemental supply, future studies incorporating sequential chemical extractions, such as exchangeable versus bound fractions, are required to quantitatively link specific geochemical fractions to temporal plant uptake rates.

### 3.2. Distinct Fates of Cerium and Europium and the Abiotic Context

A central finding of this study is the dynamic decoupling of Cerium (Ce) and Europium (Eu) anomalies across the soil–plant continuum, considered within a weakly acidic field-level soil background (mean pH 6.40 ± 0.09; Supplementary Table S2). Because the measured pH values were obtained from composite surface soil samples rather than from stage-specific bulk or rhizosphere soil samples, they provide a field-level chemical context but do not resolve temporal pH variation during soybean growth. Against this field-level geochemical background, our empirical observations demonstrate that while plant tissues consistently mirror the observed negative Eu anomaly in the soil across all developmental stages, Ce undergoes a distinct, stage-specific shift—transitioning from a soil-like negative anomaly to a pronounced positive anomaly (δCe > 1.0) in plant tissues during the peak reproductive window (45–65 days after sowing). We emphasize the imperative to strictly distinguish between this observed biogeochemical phenomenon and its hypothesized physiological mechanisms.

Mechanistically, the preferential mobilization and uptake of Ce relative to its neighboring light REEs (Lanthanum, La; Praseodymium, Pr) must be evaluated against the abiotic baseline. Under the weakly acidic field-level soil background, dissolved trivalent REE ions remain thermodynamically stable, whereas oxidized Ce tends to precipitate as insoluble tetravalent compounds (CeO_2_ or hydrous oxides) [36]. Because stage-specific bulk-soil and rhizosphere pH were not measured, the contribution of temporal soil pH variation to the observed Ce anomaly cannot be directly evaluated. We therefore propose, as a working hypothesis, that localized reduction processes at the immediate root–soil interface may contribute to the observed positive Ce anomaly shift. Specifically, active root exudation of low-molecular-weight organic acids (LMWOAs) and proton (H^+^) extrusion—coupled with localized drops in rhizosphere redox potential (Eh) driven by root respiration, microbial activity, and seasonal precipitation—thermodynamically favor the micro-environmental reduction of insoluble Ce^4+^ to the vastly more bioavailable Ce^3+^ [37]. This mobilization is further facilitated by extracellular organic ligands in root exudates that alter rare earth ion chelation and bioavailability [38]. This pathway is functionally and conceptually analogous to the “Strategy I” iron acquisition mechanisms utilized by dicotyledonous plants, which rely on localized rhizosphere acidification, organic acid exudation, and ferric reductase activity [39,40].

Because in situ rhizosphere micro-environmental parameters (pH, Eh, and LMWOAs exudation dynamics) were not directly measured in this observational study, these physiological and geochemical pathways remain working hypotheses. Future investigations utilizing in situ rhizosphere microelectrodes and transcriptomic profiling of metal transporter and reductase candidate genes are essential to strictly disentangle biological enzymatic contributions from abiotic geochemical drivers during this transitional time frame.

### 3.3. Concentration Dynamics and Phenological Synchronization

The peak in total REE concentration observed between 45 and 65 days post-sowing phenologically coincides with the transition from the flowering and pod development stages (R2–R4 stages) to the early seed-filling stages (R5–R6 stages). Here again, a clear distinction must be maintained between the empirical phenomenon and its underlying physiological drivers. Phenomenologically, the empirical data reveal a synchronized, stage-specific surge in tissue REE concentrations during peak reproductive growth, demonstrating that background REE accumulation is temporally coupled with systemic plant developmental windows during which whole-plant nutrient acquisition and metabolic demand reach their seasonal maximum [22]. Mechanistically, we hypothesize that this concentration spike reflects a transient imbalance between uptake kinetics and biomass expansion. Specifically, we propose that during this peak reproductive phase, intensified transpiration pull accelerates the mass flow of the soil solution toward the root boundary layer [41], driving a heightened bulk influx of dissolved trivalent REE ions. Consequently, we infer that the mass-flow-driven absorption rate of background REEs temporarily outpaced the concurrent “biomass dilution effect” theoretically exerted by the rapid dry matter accumulation in vegetative tissues and developing pods, a mechanistic trade-off that warrants direct experimental verification.

### 3.4. Hypotheses on Hydrodynamic and Genetic Factors Associated with Organ-Level REE Distribution

Our analytical measurements revealed markedly higher total root-associated REE concentrations than those in aerial tissues, a pattern consistent with restricted acropetal translocation to shoot tissues. However, because chemical desorption was not performed prior to root digestion, the measured root-associated pool includes both internally accumulated REEs and surface/apoplastic fractions, and the extent of true biological root sequestration cannot be quantitatively resolved. The internally retained fraction may be associated with well-documented plant defense mechanisms against trace metal accumulation, which we hypothesize to involve apoplastic cell-wall binding and vacuolar sequestration [42,43].

During the process of overcoming the root barrier for acropetal transport, the progressive increase in the LREE/HREE ratio indicates differential mobility among elements within the vascular system. This differential mobility is statistically corroborated by the inter-elemental correlations observed during the peak uptake window (Figure 4). Rather than uniform co-transport, the matrices reveal distinct element-specific couplings and decouplings across genotypes/treatments—highlighted by statistically significant positive correlations alongside significant negative correlations between specific pairs. Mechanistically, we propose that this divergent mobility stems from differences in the thermodynamic stabilities of coordination complexes formed between REE ions and endogenous organic ligands. This process is highly analogous to the long-distance vascular translocation of transition metals, which strictly relies on specialized organic chelators like nicotianamine to modulate systemic metal homeostasis [44]. Notably, as a comparable trivalent cation pathway, citric acid has been explicitly demonstrated to form stable complexes that drive the acropetal transport of nutrients within the xylem exudate of soybean [45], further supporting the role of organic acid-mediated chelation in governing the distinct hydrodynamic mobilities of LREEs and HREEs.

Furthermore, the final allocation of these translocated elements exhibits clear genotype dependency. We hypothesize that this allocation divergence may be linked to inherent differences in plant hydraulics and sink-source dynamics. Indeterminate cultivars (e.g., PA) maintain prolonged apical vegetative growth during the reproductive phase. This physiological trait theoretically sustains a more prolonged xylem transpiration stream, which could hydrodynamically favor the continuous acropetal transport of complexed REEs to aerial reproductive sinks. From a metabolic perspective, the higher seed protein accumulation inherent to the PA cultivar implies a highly active nitrogen and amino acid sink network. This intense metabolic demand might concurrently stimulate the general upregulation of endogenous metal transporters (such as the Natural Resistance-Associated Macrophage Protein (NRAMP) or Yellow Stripe-Like (YSL) families) for micronutrient mobilization, thereby facilitating the inadvertent co-transport of structurally similar REE ions into the developing grains. However, these hydrodynamic and molecular drivers remain hypotheses requiring rigorous verification through future targeted physiological and transcriptomic experiments. Conversely, the sub-determinate JY cultivar retained higher REE concentrations in its vegetative stems. These distinct patterns underscore that in-planta REE allocation is not only geochemically driven but likely regulated by biophysical and genetic factors.

### 3.5. Study Limitations and Future Perspectives

To ensure an objective interpretation of our findings, several explicit limitations of this study must be acknowledged alongside future research directions. A primary methodological constraint lies in the evaluation of root-associated REE content; because chemical desorption (e.g., via ethylenediaminetetraacetic acid (EDTA) elution) was not performed prior to digestion, the measured root REE concentrations inevitably encompass both symplastically internalized elements and fractions physically adsorbed onto the root surface or apoplast. Consequently, the markedly lower REE concentrations in aerial tissues relative to the total root-associated pool are consistent with restricted root-to-shoot translocation; however, the extent of true biological root sequestration cannot be quantitatively resolved and should therefore be interpreted with appropriate caution. Future investigations incorporating sequential chemical extractions or standardized EDTA desorption protocols are needed to strictly isolate internal biological accumulation from surface sorption.

Furthermore, a clear distinction must be maintained between the observed biogeochemical phenomena and their underlying physiological mechanisms. Although a distinct positive Ce anomaly shift was identified during the peak reproductive window, the hypothesized drivers—including root-mediated organic acid exudation, proton extrusion, and localized redox potential (Eh) drops—remain working hypotheses due to the absence of direct, in situ rhizosphere measurements. To disentangle biological enzymatic contributions from abiotic geochemical forces, future research should deploy in situ microelectrodes, rhizosphere exudate profiling, and transcriptomic analysis of key candidate transporter genes, such as members of the NRAMP and YSL families. Finally, because this study was conducted at a single experimental field site over a single growing season, inter-annual climate variations, local soil microclimates, and rhizosphere microbial dynamics could potentially influence REE bioavailability. Multi-location and multi-year field trials across diverse soil types will therefore be essential to validate the broader applicability of these baseline soil–plant REE dynamics and refine their utility as natural biogeochemical tracers in agricultural ecosystems.

## 4. Materials and Methods

### 4.1. Study Site Description

The study was conducted at the experimental agricultural base of Jilin University, located on the western periphery of Changchun, China. This site is situated within the Songnen Plain, a globally significant region of typical black soil [26]. The region is characterized by a temperate continental monsoon climate with a mean annual temperature of 7.8 °C, a frost-free period of approximately 150 days, and mean annual precipitation of ~600 mm. The soil at the experimental site is classified as black soil (Mollisol). The composite surface soil pH values, measured in a 1:2.5 (*w*/*v*) soil-to-water suspension using a glass electrode pH meter, ranged from 6.28 to 6.56 with a mean of 6.40 ± 0.09 (Supplementary Table S2). The parent material is derived from Quaternary loess and alluvial deposits overlying the Cretaceous Quantou Formation, which consists primarily of weathered sedimentary rocks such as mudstone and sandstone [27].

### 4.2. Experimental Design and Sample Collection

Two soybean cultivars with distinct genetic backgrounds and agronomic traits, ‘Jiyu 201’ (hereafter JY, sub-determinate) and ‘Ping’an 16’ (PA, indeterminate), were selected for this study. A total of 120 individuals per cultivar were initially established in experimental field plots at Jilin University. The plants were cultivated over a complete 90-day growth cycle in 2023 (sowing on 20 June 2023, following land preparation during 12–19 June 2023, and final harvest/post-harvest processing during 19–24 September 2023) under identical field conditions. A dual sampling strategy involving destructive, independent whole-plant biological replicates was employed throughout the cultivation period to capture both the temporal dynamics of REE accumulation and their final distribution in discrete plant organs. Each biological replicate represented an independent soybean plant, and destructive sampling was performed at each developmental stage; therefore, the same plant was not repeatedly sampled across different stages. The initial establishment of 120 plants per cultivar ensured sufficient material for independent destructive sampling throughout all growth stages.

First, for temporal analysis, a seven-stage sampling scheme was implemented at 10-day intervals from 20 to 80 days post-sowing, corresponding to key phenological developmental stages: Stage 2 (20 days, V2 stage), Stage 3 (30 days, V4 stage), Stage 4 (40 days, R1 flowering stage), Stage 5 (50 days, R2–R4 pod development stage), Stage 6 (60 days, R5–R6 seed-filling stage), Stage 7 (70 days, R7 maturity beginning), and Stage 8 (80 days, R8 full maturity). At each developmental stage, independent biological replicates (each representing an individual plant) were destructively harvested. Ten biological replicates per cultivar were collected for the main temporal analysis (Stages 3–7), whereas three biological replicates per cultivar were collected for Stage 2 and Stage 8 as early developmental and terminal maturity boundary references. Across the entire experiment, a total of 11 composite surface soil samples (0–20 cm depth) were prepared, and soil was not sampled separately at each developmental stage. For 10 planted locations, the pre-planting soil collected from each location was composited with the corresponding post-harvest root-zone soil from the same location, yielding 10 composite soil samples. In addition, one control composite sample was prepared by combining pre-planting soil with post-harvest soil collected from an unplanted location. Thus, a total of 11 composite soil samples were used for REE characterization and as the soil reference for subsequent soil-to-plant transfer calculations. These soil samples were not stage-specific replicates and did not correspond one-to-one with the plant samples collected at individual developmental stages. Following rigorous quality control (minimum dry matter requirements and ICP-MS matrix drift filtering) and Grubbs’ outlier testing (*p* < 0.05), final dataset sample sizes for the main temporal analysis were established as *n* = 10 for JY3–JY7 and PA4–PA5, and *n* = 6 for PA3, PA6, and PA7.

Second, for organ-level analysis, a final destructive sampling (*n* = 4 independent biological replicates) was conducted at full harvest maturity (90 days post-sowing). On this date, representative mature plants were harvested and immediately dissected into their four component organs: roots, stems, pods, and beans. These four mature plants were independent of all plants harvested during the preceding temporal sampling sequence. All collected samples were placed in polyethylene bags and transported to the laboratory for analysis.

### 4.3. Sample Pretreatment and Chemical Analysis

#### 4.3.1. Sample Pretreatment

Plant samples were sequentially rinsed with tap and deionized water, de-enzymed in an oven (105 °C, 30 min), dried to a constant weight (70 °C), and ground to pass a 200-mesh sieve. It is important to emphasize that a chemical desorption step (e.g., using EDTA or dilute acid elution) was not applied to the root samples. Consequently, the reported root REE concentrations in this study encompass both the internal symplastic fraction and those strongly adsorbed to the root surface or apoplast, representing the total root-associated REE pool. Soil samples were air-dried, cleared of debris, and ground with an agate mortar to pass a 200-mesh sieve.

Approximately 0.25 g of sieved powder (200-mesh) was digested in Teflon crucibles on a hot plate using a sequential acid mixture (HNO_3_-HF-H_2_SO_4_ followed by aqua regia). The final digestate was quantitatively transferred and diluted with ultrapure water to 25 mL, from which a 1.00 mL aliquot was further diluted to 10 mL with 1.0 mol/L HNO_3_ for analysis.

#### 4.3.2. REE Analysis and Quality Control

The concentrations of 14 REEs (La, Ce, Pr, Nd, Sm, Eu, Gd, Tb, Dy, Ho, Er, Tm, Yb, and Lu) in the final prepared solutions were determined by Inductively Coupled Plasma Mass Spectrometry (ICP-MS, NexION 350D, PerkinElmer, Waltham, MA, USA). Procedural blanks and certified reference materials (GBW07401a for soil, GBW10013 for Soybean) were processed and analyzed concurrently with the samples to ensure data quality. All analytical procedures adhered to the standards specified in the Technical Specifications for Geochemical Evaluation of Land Quality (DZ/T 0295-2016) [46]. The recovery rates for all elements were within the range of 95–105%, confirming the accuracy and reliability of the data.

#### 4.3.3. Data Analysis and Calculations

To assess REE fractionation and transfer, several geochemical parameters were calculated. First, all REE concentrations in plant and soil samples were normalized to chondrite values [33]. The magnitudes of the cerium (δCe) and europium (δEu) anomalies were calculated based on the geometric average of their chondrite-normalized neighboring elements, as follows: δCe = Ce_N_/0.5(La_N_ + Pr_N_) and δEu = Eu_N_/0.5(Sm_N_ + Gd_N_), where the subscript ‘N’ denotes the chondrite-normalized concentration of the respective element.

The soil-to-plant transfer of each REE was evaluated using a Transfer Factor (TF), defined as: TF = Cplant/Csoil, where Cplant is the concentration of an REE in the soybean sample, and Csoil is defined as the mean concentration of the corresponding REE across the 11 composite surface soil samples, which served as a common field-level soil reference rather than a stage-specific soil measurement. While samples from Stage 2 and Stage 8 were collected as baseline and maturity references, the detailed temporal analysis focuses on the critical vegetative-to-reproductive transition period (Stages 3–7). All statistical analyses—including two-tailed Student’s *t*-tests, Pearson correlation analyses, and Grubbs’ outlier tests were performed using SPSS 26.0 and Origin 2026a. All original datasets utilized for statistical analyses and data visualizations in this study, including raw elemental concentrations, calculated fractionation ratios, and descriptive statistics across all biological replicates, are comprehensively provided in Supplementary Tables S1–S18.

## 5. Conclusions

This study detailed the ‘source-to-sink’ biogeochemical distribution of REEs in a representative black soil-soybean system under background conditions. Our findings outline a clear phenomenological model characterized by: (I) a distinct temporal concentration peak between 45 and 65 days after sowing (soybean R2-R6 stages); (II) a unique shift of the Ce anomaly from negative in the soil to positive in plant tissues; and (III) distinct, genotype-dependent terminal allocation patterns, where the indeterminate growth habit is associated with greater acropetal translocation to reproductive organs. Establishing these baseline dynamics not only provides a scientific basis for deciphering the complex regulatory mechanisms of elemental homeostasis at the crop-soil interface but also lays a solid foundation for utilizing REEs as biogeochemical tracers of elemental cycling within agroecosystems in future research.

## Figures and Tables

**Table 1:** Mean relative proportions (%) of individual light and heavy rare earth elements relative to the total rare earth element concentration (ΣREE) in the surface soil samples (*n* = 11).

LREE	Proportion of ΣREE (%)	HREE	Proportion of ΣHREE (%)	Proportion of ΣREE (%)
La	17.77	Gd	38.06	4.10
Ce	36.81	Tb	4.96	0.53
Pr	4.84	Dy	21.70	2.34
Nd	24.28	Ho	4.62	0.50
Sm	4.33	Er	12.36	1.33
Eu	1.19	Tm	2.60	0.28
		Yb	13.04	1.41
		Lu	2.66	0.29
ΣLREE	89.22	ΣHREE	100.00	10.78

Note: LREE, light rare earth elements (La to Eu); HREE, heavy rare earth elements (Gd to Lu); ΣREE, total rare earth elements; ΣLREE, sum of light rare earth elements; ΣHREE, sum of heavy rare earth elements.

**Table 2:** Temporal dynamics of soil-to-plant transfer factors (TF) for 14 rare earth elements across developmental stages (Stages 3–7) in two soybean cultivars: (**a**) JY and (**b**) PA.

(**a**)					
**REEs**	**Stage 3** **V4**	**Stage 4** **R1**	**Stage 5** **R2-R4**	**Stage 6** **R5-R6**	**Stage 7** **R7**
La	0.121	0.089	0.096	0.152	0.120
Ce	0.097	0.067	0.156	0.152	0.065
Pr	0.077	0.057	0.083	0.076	0.088
Nd	0.056	0.038	0.052	0.086	0.039
Sm	0.053	0.029	0.007	0.036	0.070
Eu	0.045	0.022	0.006	0.029	0.060
Gd	0.024	0.013	0.011	0.028	0.016
Tb	0.064	0.028	0.073	0.043	0.074
Dy	0.072	0.032	0.093	0.045	0.086
Ho	0.075	0.033	0.093	0.045	0.092
Er	0.086	0.040	0.111	0.058	0.106
Tm	0.066	0.032	0.086	0.044	0.087
Yb	0.096	0.052	0.114	0.074	0.115
Lu	0.076	0.041	0.086	0.058	0.101
(**b**)					
**REEs**	**Stage 3** **V4**	**Stage 4** **R1**	**Stage 5** **R2-R4**	**Stage 6** **R5-R6**	**Stage 7** **R7**
La	0.106	0.082	0.161	0.175	0.065
Ce	0.087	0.065	0.155	0.153	0.049
Pr	0.127	0.084	0.083	0.013	0.057
Nd	0.058	0.075	0.088	0.043	0.053
Sm	0.035	0.026	0.045	0.045	0.050
Eu	0.024	0.007	0.034	0.009	0.004
Gd	0.017	0.009	0.035	0.022	0.006
Tb	0.018	0.008	0.051	0.010	0.052
Dy	0.025	0.016	0.054	0.012	0.060
Ho	0.025	0.108	0.055	0.112	0.057
Er	0.028	0.124	0.063	0.123	0.068
Tm	0.022	0.088	0.050	0.087	0.042
Yb	0.028	0.012	0.082	0.114	0.054
Lu	0.023	0.094	0.065	0.090	0.042

Note: Data are presented as mean values (*n* = 10 for JY3–JY7 and PA4–PA5; *n* = 6 for PA3, PA6, and PA7). REEs, rare earth elements; TF, transfer factor. Transfer factors were calculated as Cplant/Csoil, where Cplant is the concentration of an REE in the soybean sample, and Csoil is defined as the mean concentration of the corresponding REE across the 11 composite surface soil samples, which served as a common field-level soil reference for all developmental stages. Growth stages V4 to R7 correspond to specific soybean phenological developmental stages. The mean TF values for the two boundary stages (Stages 2 and 8) are provided in Supplementary Table S16.

## Data Availability

The datasets generated and analyzed during this study are available from the corresponding author upon reasonable request.

## Supplementary Materials

The following supporting information can be downloaded at https://www.mdpi.com/article/10.3390/plants15162485/s1.

## Abbreviations

The following abbreviations are used in this manuscript:

REEs	Rare earth elements
LREE	Light rare earth elements
HREE	Heavy rare earth elements
ΣREE	Total rare earth element concentration
ΣLREE	Sum of light rare earth element concentrations
ΣHREE	Sum of heavy rare earth element concentrations
TF	Transfer Factor
La	Lanthanum
Ce	Cerium
Pr	Praseodymium
Eu	Europium
JY	‘Jiyu 201’ cultivar
PA	‘Ping’an 16’ cultivar
ICP-MS	Inductively Coupled Plasma Mass Spectrometry
NRAMP	Natural Resistance-Associated Macrophage Protein
YSL	Yellow Stripe-Like
LMWOAs	Low-molecular-weight organic acids
Eh	Redox potential
EDTA	Ethylenediaminetetraacetic acid
SD/STD	Standard deviation
CV	Coefficient of variation
