# Supplementary material for: Dynamics of Rare Earth Element Uptake and Partitioning in Two Soybean Cultivars Under Background Soil Conditions
Source: Plants (Basel). 2026 Aug 16;15(16):2485. doi: 10.3390/plants15162485 (PMC13516251; doi:10.3390/plants15162485)
Supplement: Supplementary file 1 [file plants-15-02485-s001.zip › plants-4455819-supplementary.pdf]

Supporting Information

Dynamics of Rare Earth Element Uptake and Partitioning in Two Soybean Cultivars Under Background Soil Conditions

Yaofu Chen [1], Jilong Lu [1,*], Xinyun Zhao [1], Peng Lu [1], Jiaxuan Cui [1], Kaiyu Zhang [1], Jiayu Qu [1] and Yaru Hou [2]

1 College of Geoexploration Science and Technology, Jilin University, Changchun 130026, China

2 School of Geomatics and Prospecting Engineering, Jilin Jianzhu University, Changchun, 130118, China

* Correspondence: lujl@jlu.edu.cn; Tel.: +86-130-7430-6176

Supplementary Table S1. Summary statistics (minimum, maximum, average, standard deviation, and coefficient of variation) of total rare earth element concentrations and associated fractionation ratios in 11 soil samples.

Sample		ΣREE (mg/kg)	ΣLREE (mg/kg)	ΣHREE (mg/kg)	ΣLREE/ΣHREE	La/Yb	La/Sm	Gd/Yb
Soil 1 to	Min	43.44	38.23	5.22	5.82	9.04	3.40	2.12
Soil 11	Max	245.91	212.06	33.85	13.29	19.74	4.88	4.46
	Avg	130.24	116.19	14.04	8.44	12.9	4.08	2.95
	STD	67.78	60.61	8.13	2.10	3.34	0.45	0.74
	CV	52.04%	52.17%	57.89%	24.91%	25.86%	11.12%	25.04%

Note: ΣREE, total rare earth elements; ΣLREE, light rare earth elements; ΣHREE, heavy rare earth elements. STD, standard deviation; CV, coefficient of variation. Concentrations are expressed in mg/kg. Ratios such as La/Yb, La/Sm, and Gd/Yb represent chondrite-normalized values.

Supplementary Table S2. Raw concentrations of 14 individual rare earth elements (La to Lu) and measured pH values in eleven soil samples (Soil 1 – Soil 11).

Soil Sample	La	Ce	Pr	Nd	Sm	Eu	Gd
Soil 1	26.51	43.64	7.74	34.15	7.15	1.94	6.96
Soil 2	15.77	26.17	3.89	18.3	3.23	0.92	2.72
Soil 3	20.72	84.36	5.37	42.39	5.19	1.23	4.91

Soil 4	24.16	52.51	6.43	33.15	5.88	1.71	5.12
Soil 5	49.27	74.59	13.82	58.76	12.51	3.11	11.78
Soil 6	36.71	70.94	8.69	46.22	7.85	2.08	7.23
Soil 7	37.46	80.63	10.34	52.64	8.69	2.33	9.5
Soil 8	7.6	15.02	2.37	10.73	1.92	0.59	1.63
Soil 9	10.21	29.41	3.26	17.64	2.87	0.74	2.92
Soil 10	11.75	24.84	3.58	16.56	3.46	1.5	3.03
Soil 11	14.37	25.29	3.85	17.22	3.29	0.91	2.99

Soil Sample	Tb	Dy	Ho	Er	Tm	Yb	Lu	Soil pH
Soil 1	0.93	4.42	0.81	1.92	0.3	2.04	0.31	6.48
Soil 2	0.36	1.97	0.4	1.15	0.21	1.2	0.21	6.36
Soil 3	0.51	2.48	0.55	1.61	0.22	1.53	0.17	6.45
Soil 4	0.67	2.94	0.59	1.7	0.3	1.86	0.3	6.28
Soil 5	1.43	7.22	1.57	4.55	0.84	5.45	1.01	6.41
Soil 6	0.66	3.6	0.64	1.87	0.42	1.86	0.39	6.45
Soil 7	0.96	4.34	0.73	2.29	0.47	2.13	0.4	6.30
Soil 8	0.36	1.1	0.33	0.73	0.15	0.77	0.15	6.32
Soil 9	0.41	1.57	0.29	0.97	0.14	1.07	0.22	6.56
Soil 10	0.89	1.96	0.83	1.29	0.71	1.14	0.75	6.49
Soil 11	0.48	1.92	0.39	1.01	0.25	1.09	0.21	6.33

Note: Concentrations are expressed in mg/kg. Soil pH was measured in a 1:2.5 (w/v) soil-to-water suspension using a glass electrode pH meter.

Supplementary Table S3. Raw concentrations of 14 individual rare earth elements (La to Lu) in ten replicate samples (S1 – S10) of the 'Jiyu 201' (JY) soybean cultivar at developmental stage 3 (JY3).

JY3 Sample	La	Ce	Pr	Nd	Sm	Eu	Gd
S1	2.62	5	0.44	1.73	0.22	0.07	0.12
S2	2.86	5.08	0.5	1.83	0.31	0.08	0.14
S3	3.16	4.34	0.51	1.51	0.36	0.07	0.1
S4	2.79	4.57	0.45	1.96	0.39	0.1	0.16
S5	2.4	3.95	0.5	2	0.32	0.04	0.13
S6	2.95	4.18	0.43	1.65	0.29	0.07	0.11
S7	3.08	4.93	0.44	1.79	0.3	0.07	0.13
S8	2.66	4.5	0.45	1.86	0.29	0.05	0.19
S9	2.99	4.61	0.56	1.58	0.19	0.08	0.13
S10	2.49	5.24	0.52	1.69	0.33	0.07	0.09

JY3 Sample	Tb	Dy	Ho	Er	Tm	Yb	Lu

S1	0.05	0.18	0.05	0.15	0.02	0.18	0.03
S2	0.04	0.23	0.05	0.16	0.02	0.22	0.04
S3	0.04	0.22	0.05	0.15	0.02	0.18	0.03
S4	0.04	0.24	0.05	0.13	0.02	0.15	0.03
S5	0.04	0.19	0.05	0.15	0.02	0.17	0.03
S6	0.04	0.22	0.05	0.15	0.02	0.17	0.03
S7	0.03	0.23	0.04	0.15	0.02	0.19	0.03
S8	0.04	0.22	0.05	0.12	0.02	0.18	0.03
S9	0.04	0.24	0.06	0.18	0.02	0.18	0.03
S10	0.04	0.23	0.05	0.16	0.02	0.18	0.02

Note: Concentrations are expressed in mg/kg. JY3 denotes the third sampling stage.

Supplementary Table S4. Raw concentrations of 14 individual rare earth elements (La to Lu) in six replicate samples (S1 – S6) of the 'Ping'an 16' (PA) soybean cultivar at developmental stage 3 (PA3).

PA3 Sample	La	Ce	Pr	Nd	Sm	Eu	Gd
S1	2.26	3.51	0.89	1.73	0.21	0.04	0.06
S2	2.84	4.82	0.92	2.16	0.25	0.028	0.07
S3	2.65	3.95	0.76	1.51	0.16	0.037	0.09
S4	2.4	4.34	0.68	1.82	0.17	0.033	0.1
S5	2.55	3.86	0.71	1.79	0.23	0.058	0.13

S6	2	4.66	0.84	1.97	0.18	0.044	0.09

PA3 Sample	Tb	Dy	Ho	Er	Tm	Yb	Lu
S1	0.01	0.09	0.02	0.05	0.01	0.05	0.01
S2	0.01	0.07	0.02	0.05	0.01	0.06	0.01
S3	0.01	0.06	0.02	0.06	0.01	0.05	0.01
S4	0.01	0.09	0.02	0.04	0.01	0.04	0.01
S5	0.01	0.08	0.02	0.05	0.01	0.05	0.01
S6	0.01	0.09	0.02	0.05	0.01	0.05	0.01

Note: Concentrations are expressed in mg/kg. PA3 denotes the third sampling stage.

Supplementary Table S5. Raw concentrations of 14 individual rare earth elements (La to Lu) in ten replicate samples (S1 – S10) of the 'Jiyu 201' (JY) soybean cultivar at developmental stage 4 (JY4).

JY4 Sample	La	Ce	Pr	Nd	Sm	Eu	Gd
S1	1.88	2.9	0.33	1.03	0.18	0.03	0.06
S2	1.93	3.74	0.41	1.33	0.16	0.032	0.11
S3	2.11	3.45	0.4	1.34	0.17	0.03	0.07
S4	1.7	3.52	0.38	1.39	0.19	0.027	0.06
S5	2.42	3	0.39	1.26	0.17	0.032	0.05

S6	2.29	2.84	0.36	1.25	0.15	0.03	0.09
S7	2.2	3.22	0.33	1.18	0.2	0.023	0.08
S8	2.12	3.18	0.33	1.11	0.17	0.04	0.06
S9	2.03	2.76	0.3	1.07	0.14	0.026	0.04
S10	1.92	3.29	0.37	1.14	0.17	0.03	0.08

JY4 Sample	Tb	Dy	Ho	Er	Tm	Yb	Lu
S1	0.02	0.11	0.03	0.07	0.01	0.11	0.02
S2	0.02	0.1	0.02	0.07	0.01	0.11	0.02
S3	0.02	0.1	0.02	0.08	0.01	0.08	0.02
S4	0.02	0.1	0.02	0.07	0.01	0.1	0.02
S5	0.02	0.11	0.02	0.07	0.01	0.11	0.03
S6	0.03	0.1	0.02	0.07	0.01	0.11	0.02
S7	0.02	0.12	0.02	0.08	0.01	0.1	0.02
S8	0.02	0.07	0.01	0.07	0.01	0.1	0.02
S9	0.02	0.09	0.02	0.05	0.01	0.09	0.02
S10	0.01	0.1	0.02	0.07	0.01	0.09	0.01

Note: Concentrations are expressed in mg/kg. JY4 denotes the fourth sampling stage.

Supplementary Table S6. Raw concentrations of 14 individual rare earth elements (La to Lu) in ten replicate samples (S1 ‑ S10) of the 'Ping'an 16' (PA) soybean cultivar at developmental stage 4 (PA4).

PA4 Sample	La	Ce	Pr	Nd	Sm	Eu	Gd
S1	1.62	3.79	0.45	2.61	0.11	0.007	0.02
S2	1.95	2.97	0.52	2.19	0.16	0.008	0.05
S3	1.93	2.56	0.49	2.01	0.17	0.005	0.04
S4	2.2	2.68	0.57	2.34	0.16	0.01	0.05
S5	1.7	3.17	0.43	2.42	0.13	0.01	0.06
S6	1.94	3.41	0.62	2.1	0.13	0.016	0.03
S7	1.67	3.31	0.52	2.48	0.14	0.01	0.05
S8	2.16	2.75	0.54	2.77	0.14	0.012	0.07
S9	1.61	3.23	0.53	2.75	0.21	0.013	0.08
S10	2.12	3.13	0.63	2.03	0.15	0.009	0.05

PA4 Sample	Tb	Dy	Ho	Er	Tm	Yb	Lu
S1	0.01	0.05	0.07	0.19	0.03	0.02	0.03
S2	0.01	0.04	0.08	0.19	0.04	0.01	0.05
S3	0.01	0.05	0.07	0.28	0.03	0.02	0.04
S4	0.01	0.05	0.08	0.22	0.03	0.02	0.04

S5	0.01	0.06	0.06	0.22	0.02	0.02	0.04
S6	0.01	0.04	0.06	0.2	0.03	0.02	0.04
S7	0.01	0.05	0.06	0.23	0.03	0.02	0.04
S8	0.01	0.06	0.06	0.24	0.03	0.03	0.03
S9	0.01	0.05	0.08	0.19	0.03	0.02	0.05
S10	0.01	0.05	0.08	0.24	0.03	0.02	0.04

Note: Concentrations are expressed in mg/kg. PA4 denotes the fourth sampling stage.

Supplementary Table S7. Raw concentrations of 14 individual rare earth elements (La to Lu) in ten replicate samples (S1 – S10) of the 'Jiyu 201' (JY) soybean cultivar at developmental stage 5 (JY5).

JY5 Sample	La	Ce	Pr	Nd	Sm	Eu	Gd
S1	2.1	6.87	0.53	1.75	0.06	0.006	0.06
S2	1.92	6.89	0.55	1.54	0.04	0.012	0.03
S3	2.31	8.49	0.44	1.63	0.05	0.008	0.07
S4	2.13	6.77	0.51	1.63	0.03	0.01	0.06
S5	2.17	6.98	0.5	1.84	0.04	0.013	0.02
S6	2.64	7.22	0.5	1.65	0.06	0.01	0.05
S7	2.18	7.03	0.53	1.55	0.04	0.01	0.06
S8	2.41	8.07	0.61	1.32	0.02	0.007	0.09
S9	2.07	8.03	0.56	1.84	0.04	0.014	0.1

S10	2.37	8.45	0.47	1.65	0.02	0.01	0.06

JY5 Sample	Tb	Dy	Ho	Er	Tm	Yb	Lu
S1	0.06	0.29	0.07	0.17	0.04	0.2	0.02
S2	0.06	0.25	0.06	0.18	0.02	0.22	0.04
S3	0.05	0.24	0.06	0.22	0.03	0.25	0.03
S4	0.08	0.27	0.05	0.19	0.03	0.19	0.03
S5	0.05	0.26	0.05	0.19	0.03	0.22	0.04
S6	0.04	0.31	0.07	0.18	0.02	0.22	0.03
S7	0.05	0.3	0.06	0.17	0.03	0.18	0.03
S8	0.02	0.25	0.06	0.22	0.02	0.21	0.03
S9	0.04	0.31	0.06	0.2	0.04	0.2	0.02
S10	0.05	0.32	0.06	0.18	0.04	0.21	0.03

Note: Concentrations are expressed in mg/kg. JY5 denotes the fifth sampling stage.

Supplementary Table S8. Raw concentrations of 14 individual rare earth elements (La to Lu) in ten replicate samples (S1 – S10) of the 'Ping'an 16' (PA) soybean cultivar at developmental stage 5 (PA5).

PA5 Sample	La	Ce	Pr	Nd	Sm	Eu	Gd
S1	4.07	6.95	0.53	2.49	0.29	0.05	0.16

S2	3.79	6.63	0.49	2.95	0.26	0.06	0.17
S3	3.92	7.89	0.56	2.76	0.25	0.07	0.24
S4	4.17	8.16	0.49	3.1	0.26	0.05	0.19
S5	3.76	6.89	0.58	2.59	0.28	0.04	0.19
S6	3.28	8.12	0.51	2.64	0.2	0.05	0.22
S7	3.51	7.49	0.48	2.57	0.23	0.03	0.16
S8	3.31	8.03	0.59	2.72	0.25	0.05	0.18
S9	3.32	7.67	0.51	2.95	0.21	0.04	0.21
S10	4.07	6.47	0.46	3.13	0.27	0.06	0.18

PA5 Sample	Tb	Dy	Ho	Er	Tm	Yb	Lu
S1	0.04	0.14	0.042	0.14	0.02	0.16	0.025
S2	0.041	0.2	0.04	0.13	0.018	0.16	0.014
S3	0.045	0.19	0.037	0.11	0.022	0.15	0.02
S4	0.04	0.12	0.036	0.11	0.02	0.12	0.021
S5	0.042	0.16	0.036	0.09	0.025	0.14	0.02
S6	0.04	0.15	0.035	0.11	0.02	0.16	0.018
S7	0.037	0.19	0.042	0.09	0.017	0.16	0.016
S8	0.035	0.16	0.048	0.11	0.015	0.14	0.02
S9	0.04	0.16	0.04	0.1	0.02	0.16	0.024
S10	0.04	0.13	0.044	0.11	0.023	0.15	0.022

Note: Concentrations are expressed in mg/kg. PA5 denotes the fifth sampling stage.

Supplementary Table S9. Raw concentrations of 14 individual rare earth elements (La to Lu) in ten replicate samples (S1 – S10) of the 'Jiyu 201' (JY) soybean cultivar at developmental stage 6 (JY6).

JY6 Sample	La	Ce	Pr	Nd	Sm	Eu	Gd
S1	3.56	6.87	0.5	2.34	0.27	0.04	0.18
S2	3.29	7.07	0.42	2.59	0.18	0.05	0.12
S3	3.33	7.79	0.59	2.31	0.21	0.04	0.13
S4	4.02	6.19	0.37	2.63	0.23	0.05	0.17
S5	3.18	8.37	0.54	2.85	0.2	0.03	0.1
S6	3.12	7.9	0.47	2.91	0.23	0.05	0.13
S7	3.92	5.88	0.48	3.15	0.18	0.04	0.19
S8	4.18	7.47	0.49	3.15	0.22	0.03	0.17
S9	2.74	6.55	0.51	2.18	0.21	0.04	0.14
S10	3.76	8.81	0.43	2.99	0.17	0.03	0.17

JY6 Sample	Tb	Dy	Ho	Er	Tm	Yb	Lu
S1	0.03	0.14	0.02	0.12	0.02	0.15	0.02

S2	0.03	0.11	0.03	0.1	0.02	0.12	0.01
S3	0.03	0.14	0.03	0.08	0.02	0.13	0.02
S4	0.04	0.13	0.04	0.1	0.02	0.15	0.02
S5	0.03	0.15	0.03	0.1	0.02	0.13	0.02
S6	0.03	0.12	0.03	0.1	0.02	0.14	0.03
S7	0.03	0.13	0.03	0.12	0.01	0.12	0.02
S8	0.03	0.15	0.03	0.1	0.03	0.12	0.02
S9	0.03	0.17	0.03	0.1	0.02	0.16	0.02
S10	0.02	0.16	0.03	0.08	0.02	0.18	0.02

Note: Concentrations are expressed in mg/kg. JY6 denotes the sixth sampling stage.

Supplementary Table S10. Raw concentrations of 14 individual rare earth elements (La to Lu) in six replicate samples (S1 – S6) of the 'Ping'an 16' (PA) soybean cultivar at developmental stage 6 (PA6).

PA6 Sample	La	Ce	Pr	Nd	Sm	Eu	Gd
S1	4.2	7.74	0.08	1.66	0.24	0.016	0.07
S2	3.82	5.78	0.05	1.43	0.25	0.009	0.14
S3	3.61	8.19	0.09	1.13	0.21	0.012	0.11
S4	4.54	8.71	0.09	1.26	0.22	0.009	0.12
S5	3.27	7.12	0.08	1.16	0.26	0.006	0.12
S6	4.92	6.44	0.09	1.46	0.32	0.008	0.16

PA6 Sample	Tb	Dy	Ho	Er	Tm	Yb	Lu
S1	0.01	0.04	0.05	0.17	0.03	0.17	0.04
S2	0.01	0.05	0.07	0.25	0.02	0.18	0.03
S3	0.01	0.03	0.08	0.17	0.03	0.27	0.03
S4	0.01	0.04	0.07	0.23	0.03	0.21	0.02
S5	0.01	0.04	0.07	0.22	0.03	0.23	0.03
S6	0.01	0.04	0.08	0.22	0.04	0.2	0.03

Note: Concentrations are expressed in mg/kg. PA6 denotes the sixth sampling stage.

Supplementary Table S11. Raw concentrations of 14 individual rare earth elements (La to Lu) in ten replicate samples (S1 – S10) of the 'Jiyu 201' (JY) soybean cultivar at developmental stage 7 (JY7).

JY7 Sample	La	Ce	Pr	Nd	Sm	Eu	Gd
S1	2.6	2.5	0.57	1.06	0.32	0.13	0.11
S2	2.71	3.13	0.53	1.13	0.39	0.07	0.1
S3	2.99	3.03	0.59	1.1	0.44	0.09	0.09
S4	2.48	3.43	0.68	1.41	0.35	0.08	0.05
S5	2.85	3.46	0.56	1.32	0.41	0.1	0.09
S6	3.11	3.15	0.56	1.36	0.46	0.09	0.08

S7	2.39	2.78	0.52	1.3	0.48	0.07	0.13
S8	2.84	3.22	0.5	1.09	0.34	0.1	0.07
S9	2.85	3.17	0.6	1.16	0.36	0.06	0.1
S10	2.98	3.33	0.5	1.27	0.35	0.11	0.08

JY7 Sample	Tb	Dy	Ho	Er	Tm	Yb	Lu
S1	0.05	0.33	0.06	0.15	0.03	0.22	0.04
S2	0.07	0.25	0.06	0.2	0.03	0.22	0.04
S3	0.05	0.26	0.06	0.16	0.03	0.21	0.05
S4	0.06	0.27	0.05	0.21	0.03	0.22	0.04
S5	0.04	0.28	0.05	0.18	0.03	0.2	0.04
S6	0.05	0.21	0.08	0.18	0.03	0.22	0.04
S7	0.05	0.26	0.06	0.19	0.02	0.16	0.04
S8	0.03	0.24	0.06	0.15	0.03	0.2	0.04
S9	0.05	0.21	0.06	0.17	0.03	0.22	0.04
S10	0.05	0.29	0.06	0.21	0.04	0.23	0.03

Note: Concentrations are expressed in mg/kg. JY7 denotes the seventh sampling stage.

Supplementary Table S12. Raw concentrations of 14 individual rare earth elements (La to Lu) in six replicate samples (S1 – S6) of the 'Ping'an 16' (PA) soybean cultivar at developmental stage 7 (PA7).

PA7 Sample	La	Ce	Pr	Nd	Sm	Eu	Gd
S1	1.68	2.57	0.35	1.44	0.22	0.012	0.02
S2	1.57	2.68	0.35	1.86	0.28	0.01	0.028
S3	1.49	2.32	0.29	1.92	0.36	0.008	0.04
S4	1.25	2.26	0.41	1.59	0.21	0.007	0.03
S5	1.4	2.16	0.36	1.73	0.32	0.014	0.024
S6	1.67	1.99	0.4	1.54	0.29	0.009	0.038

PA7 Sample	Tb	Dy	Ho	Er	Tm	Yb	Lu
S1	0.04	0.2	0.03	0.1	0.02	0.1	0.02
S2	0.05	0.17	0.05	0.12	0.02	0.11	0.02
S3	0.04	0.17	0.04	0.11	0.02	0.11	0.02
S4	0.03	0.19	0.04	0.14	0.02	0.11	0.02
S5	0.04	0.2	0.04	0.11	0.02	0.07	0.02
S6	0.04	0.15	0.04	0.14	0.02	0.1	0.02

Note: Concentrations are expressed in mg/kg. PA7 denotes the seventh sampling stage.

Supplementary Table S13. Summary statistics (minimum, maximum, average, standard deviation, and coefficient of variation) of total rare earth element concentrations and associated fractionation ratios in the 'Jiyu 201' (JY) soybean cultivar across seven developmental stages (JY2 – JY8).

Sample		ΣREE (mg/kg)	ΣLREE (mg/kg)	ΣHREE (mg/kg)	ΣLREE/ΣHREE	La/Yb	La/Sm	Gd/Yb
JY2 to JY8	Min	7.43	7.02	0.41	8.87	10.62	7.13	0.29
	Max	14.87	14.24	0.92	22.6	25.07	55.75	1.07
	Avg	10.85	10.15	0.7	15.4	17.75	17.94	0.65
	STD	2.48	2.41	0.21	4.71	5.01	16.96	0.26
	CV	22.84%	23.78%	29.51%	30.59%	28.24%	94.53%	39.65%

Note: ΣREE, total rare earth elements; ΣLREE, light rare earth elements; ΣHREE, heavy rare earth elements. STD, standard deviation; CV, coefficient of variation. Concentrations are expressed in mg/kg. Ratios such as La/Yb, La/Sm, and Gd/Yb represent chondrite-normalized values.

Supplementary Table S14. Summary statistics (minimum, maximum, average, standard deviation, and coefficient of variation) of total rare earth element concentrations and associated fractionation ratios in the 'Ping'an 16' (PA) soybean cultivar across seven developmental stages (PA2–PA8).

Sample	ΣREE (mg/kg)	ΣLREE (mg/kg)	ΣHREE (mg/kg)	ΣLREE/ΣHREE	La/Yb	La/Sm	Gd/Yb

PA2 to PA8	Min	6.72	6.17	0.32	11.01		11.29	4.92	0.24
	Max	15.49	14.76	0.73	29.72		94.5	16.24	2.5
	Avg	10.51	9.93	0.58	17.93		35.33	11.7	1.26
	STD	3.09	3.02	0.15	6.32		29	4.71	0.91
	CV	29.41%	30.39%	26.62%	35.28%		82.09%	40.24%	72.80%

Note: ΣREE, total rare earth elements; ΣLREE, light rare earth elements; ΣHREE, heavy rare earth elements. STD, standard deviation; CV, coefficient of variation. Concentrations are expressed in mg/kg. Ratios such as La/Yb, La/Sm, and Gd/Yb represent chondrite-normalized values.

Supplementary Table S15. Average concentrations of 14 individual rare earth elements (La to Lu) in the 'Jiyu 201' (JY) and 'Ping'an 16' (PA) soybean cultivars at developmental stages 2 and 8.

Sample	La	Ce	Pr	Nd	Sm	Eu	Gd
JY2	3.21	4.62	0.6	1.79	0.3	0.07	0.1
PA2	2.66	5.98	0.2	0.87	0.17	0.04	0.17
JY8	2.35	4.48	0.34	1.7	0.17	0.04	0.09
PA8	1.92	2.97	0.5	2.25	0.39	0.01	0.04

Sample	Tb	Dy	Ho	Er	Tm	Yb	Lu
JY2	0.04	0.2	0.04	0.14	0.02	0.18	0.03
PA2	0.02	0.13	0.03	0.08	0.01	0.08	0.01

JY8	0.02	0.11	0.02	0.08	0.01	0.11	0.02
PA8	0.04	0.23	0.05	0.15	0.02	0.17	0.03

Note: Concentrations are expressed in mg/kg. The values represent the average of three biological replicates (*n* = 3) for each sample. JY2 and PA2 denote the second sampling stage, while JY8 and PA8 denote the eighth sampling stage

Supplementary Table S16. Soil-to-plant transfer factors (TF) of 14 individual rare earth elements (La to Lu) in the 'Jiyu 201' (JY) and 'Ping'an 16' (PA) soybean cultivars at developmental Stages 2 and 8.

Sample	La	Ce	Pr	Nd	Sm	Eu	Gd
JY2	0.139	0.096	0.096	0.057	0.054	0.047	0.019
PA2	0.115	0.125	0.031	0.027	0.031	0.025	0.033
JY8	0.101	0.093	0.054	0.054	0.031	0.025	0.017
PA8	0.083	0.062	0.080	0.071	0.068	0.005	0.007

Sample	Tb	Dy	Ho	Er	Tm	Yb	Lu
JY2	0.056	0.067	0.068	0.083	0.064	0.096	0.079
PA2	0.033	0.041	0.040	0.044	0.032	0.041	0.030
JY8	0.034	0.036	0.037	0.046	0.036	0.059	0.047
PA8	0.064	0.075	0.079	0.089	0.067	0.092	0.067

Note: Soil-to-plant transfer factors (TF) were calculated as the ratio of REE concentrations in soybean tissues to the average REE concentrations in surface soil samples (TF = Cplant/Csoil).

The values represent the mean TF values calculated from three independent biological replicates (n = 3) for each cultivar at each developmental stage. Stage 2 and Stage 8 were included as early developmental baseline and terminal maturity references, respectively, whereas detailed temporal TF dynamics were evaluated during Stages 3–7 using larger biological replication.

Supplementary Table S17. Raw concentrations of total rare earth elements ΣREE in discrete organs (roots, stems, pods, and beans) of 'Jiyu 201' (JY) and 'Ping'an 16' (PA) mature soybean plants across four biological replicates (S1 – S4).

Sample	JY Roots	PA Roots	JY Stems	PA Stems	JY Pods	PA Pods	JY Beans	PA Beans
S1	17.07	26.52	108.45	60.35	40.11	67.89	63.94	115.68
S2	18.44	29.45	120.37	68.12	44.12	75.46	68.45	136.42
S3	20.23	21.29	138.92	76.88	47.88	91.3	76.89	165.4
S4	20.02	27.26	151.02	84.65	52.81	98.87	81.4	186.14

Note: ΣREE, total rare earth elements. S1–S4 represent four independent biological replicates sampled at full harvest maturity. Due to the massive concentration gradient across the root barrier, the concentration unit for roots is mg/kg, whereas for aerial organs (stems, pods, and beans), it is expressed in **μ**g/kg.

Supplementary Table S18. Average concentrations of total, light, and heavy rare earth elements and their specific fractionation ratios in discrete organs (roots, stems, pods, and beans) of two mature soybean cultivars ('Jiyu 201' and 'Ping'an 16').

Sample	ΣREE	ΣLREE	ΣHREE	ΣLREE/ΣHREE	La/Yb	La/Sm	Gd/Yb
JY Roots	18.94	17.6	1.34	13.13	20.14	4.34	3.93
JY Stems	129.69	114.08	15.61	7.31	5.00	5.19	0.77
JY Pods	46.23	44.04	2.19	20.11	22.28	14.85	0.88
JY Beans	72.67	69.64	3.03	22.98	25.43	17.04	1.04
PA Roots	26.13	23.13	3	7.71	20.25	4.50	9.15
PA Stems	72.5	68.93	3.57	19.31	23.96	13.33	1.05
PA Pods	83.38	80.49	2.89	27.85	163.90	36.62	6.76
PA Beans	150.91	144.33	6.58	21.93	22.72	16.04	0.87

Note: ΣREE, total rare earth elements; ΣLREE, light rare earth elements; ΣHREE, heavy rare earth elements. Due to the large concentration gradient across the root barrier, the concentration unit for roots is mg/kg, whereas for aerial organs (stems, pods, and beans), it is expressed in **μ**g/kg. Ratios such as La/Yb, La/Sm, and Gd/Yb represent chondrite-normalized values.
